# Association of polygenic liabilities for schizophrenia and bipolar disorder with educational attainment and cognitive aging

**DOI:** 10.1038/s41398-024-03182-6

**Published:** 2024-11-16

**Authors:** Chi-Shin Wu, Chia-Lin Hsu, Mei-Chen Lin, Mei-Hsin Su, Yen-Feng Lin, Chia-Yen Chen, Po-Chang Hsiao, Yi-Jiun Pan, Pei-Chun Chen, Yen-Tsung Huang, Shi-Heng Wang

**Affiliations:** 1 National Center for Geriatrics and Welfare Research, National Health Research Institutes, Zhunan, Taiwan; 2 College of Public Health, China Medical University, Taichung, Taiwan; 3 Department of Psychiatry, Virginia Institute for Psychiatric Behavioral Genetics, Virginia Commonwealth University, Richmond, VA USA; 4 Center for Neuropsychiatric Research, National Health Research Institutes, Miaoli, Taiwan; 5 Biogen, Cambridge, MA USA; 6 Stanley Center for Psychiatric Research, Broad Institute of MIT and Harvard, Cambridge, MA USA; 7 College of Public Health, National Taiwan University, Taipei, Taiwan; 8 School of Medicine, College of Medicine, China Medical University, Taichung, Taiwan; 9 Institute of Statistical Science, Academia Sinica, Taipei, Taiwan; 10 Department of Medical Research, China Medical University Hospital, China Medical University, Taichung, Taiwan

**Keywords:** Clinical genetics, Schizophrenia

## Abstract

To elucidate the specific and shared genetic background of schizophrenia (SCZ) and bipolar disorder (BPD), this study explored the association of polygenic liabilities for SCZ and BPD with educational attainment and cognitive aging. Among 106,806 unrelated community participants from the Taiwan Biobank, we calculated the polygenic risk score (PRS) for SCZ (PRS_SCZ_) and BPD (PRS_BPD_), shared PRS between SCZ and BPD (PRS_SCZ+BPD_), and SCZ-specific PRS (PRS_SCZvsBPD_). Based on the sign-concordance of the susceptibility variants with SCZ/BPD, PRS_SCZ_ was split into PRS_SCZ_concordant_/PRS_SCZ_discordant_, and PRS_BPD_ was split into PRS_BPD_concordant_/PRS_BPD_discordant_. Ordinal logistic regression models were used to estimate the association with educational attainment. Linear regression models were used to estimate the associations with cognitive aging (*n* = 27,005), measured by the Mini-Mental State Examination (MMSE), and with MMSE change (*n* = 6194 with mean follow-up duration of 3.9 y) in individuals aged≥ 60 years. PRS_SCZ,_ PRS_BPD_, and PRS_SCZ+BPD_ were positively associated with educational attainment, whereas PRS_SCZvsBPD_ was negatively associated with educational attainment. PRS_SCZ_ was negatively associated with MMSE, while PRS_BPD_ was positively associated with MMSE. The concordant and discordant parts of polygenic liabilities have contrasting association, PRS_SCZ_concordant_ and PRS_BPD_concordant_ mainly determined these effects mentioned above_._ PRS_SCZvsBPD_ predicted decreases in the MMSE scores. Using a large collection of community samples, this study provided evidence for the contrasting effects of polygenic architecture in SCZ and BPD on educational attainment and cognitive aging and suggested that SCZ and BPD were not genetically homogeneous.

## Introduction

Major psychiatric disorders are differentiated based on symptom patterns; however, clinical features, such as psychosis and cognitive impairment, have also been known to transcend diagnostics. The overlapping psychopathology implies there might be shared causative processes; the boundaries between psychiatric disorders are not clear-cut [1, 2]. Indeed, over 100 genome-wide significant loci shared between schizophrenia (SCZ) and bipolar disorder (BPD) have been identified [3]. Using a polygenic risk score approach to explore genetic overlap [4], a cross-disorder association between SCZ and BPD was revealed [5]. Beyond shared polygenic risk, a recent large genome-wide association study (GWAS) further explored the shared symptoms between SCZ and BPD driven by the same underlying polygenic profiling [3].

Cognitive deficits are the core symptoms of SCZ [6–9], usually occur many years before the onset of SCZ [6, 10–12], and may worsen after psychosis is present [6]. Cognitive function also declines in patients with BPD, although the magnitude of the decline in cognition is lesser in patients with BPD than in patients with SCZ [13–15]. Cognitive impairment might have different manifestations over the natural disease course. Early onset of these diseases may interrupt the educational process, resulting in lower educational attainment [16–18]. Cognition impairment in adulthood might increase the risk of developing dementia [19, 20].

However, recent studies showed that general people with higher polygenic liabilities for SCZ or BPD have higher educational attainment [21, 22]. Genetic studies using GWAS summary data demonstrated the coefficient of correlation with education is stronger for the polygenic liabilities of BPD than SCZ [23, 24]. In addition, the associations between polygenic liabilities of SCZ and educational attainment were inconsistent in other studies [23, 25]. GWAS has identified specific loci for distinguishing between SCZ and BPD [3]; the SCZ-specific genetic background differentiated from BPD has a negative genetic correlation with educational attainment [23]. The polygenic liabilities of SCZ or BPD might play a different role on cognitive performance in adulthood. Several genetic loci related to SCZ are associated with poor cognitive performance [25]. A higher polygenic risk for SCZ is associated with lower cognitive performance in the general population [22, 26–29] and clinical samples [29–31]. A Mendelian randomization analysis showed that there is a bidirectional causal association between SCZ and intelligence; in contrast, there is no significant causal association between BPD and intelligence [32]. A negative correlation between general cognitive function and genetic factors differentiating SCZ from BPD has been revealed using GWAS summary data [23]. Currently, the genetic evidence for the association between SCZ/BPD and cognitive aging in late adulthood is limited. It is unclear whether polygenic liabilities for SCZ and BPD and their shared and specific effects are associated with cognitive aging.

Based on the heterogeneous effect of genetic background for SCZ on cognition, it has been proposed that the diagnosis of SCZ gathers at least two disease subtypes [33]. One subtype is similar to BPD and has high intelligence, and the other who is independent of BPD manifests cognitive impairment. To elucidate the genetic background of SCZ and BPD and better understand the nosology of psychiatric disorders, this study aimed to explore the shared and specific effects of polygenic liabilities for SCZ and BPD on educational attainment and cognitive aging among a large collection of community samples from the Taiwan Biobank. In addition, we used the sign-concordance of the susceptibility variants with SCZ/BPD, and polygenic liabilities of SCZ and BPD were split into concordant and discordant parts, respectively. We hypothesize that (1) polygenic liabilities for SCZ and BPD and their shared effects were associated with higher educational attainment, and SCZ-specific polygenic liabilities differentiated from BPD was associated with lower educational attainment. (2) Polygenic liabilities for SCZ and BPD were associated with elevated and lower, respectively, risk for cognitive aging, and SCZ-specific polygenic liabilities differentiated from BPD was associated with elevated risk for cognitive aging. (3) The concordant and discordant parts of polygenic liabilities have contrasting association with educational attainment and cognitive aging, which provided ecidence for that SCZ and BPD were not genetically homogeneous.

## Materials and methods

### Study samples and measurements

The study participants were recruited from the Taiwan Biobank [34, 35], the largest government-supported biobank in Taiwan since 2012, a prospective cohort study with genomic data and repeated measurements of a wide range of phenotypes collected, with an expected final sample size of 200,000. Repeated measurements of phenotypes are planned to be followed up every 2–4 years. The Taiwan Biobank recruits community-based participants aged 30–70 years without a history of cancer. The recruitment and sample collection procedures were approved by the internal review board of the Taiwan Biobank. Each participant signed an approved informed consent form, provided blood samples, and participated in face-to-face interviews. This study was approved by the Central Regional Research Ethics Committee of the China Medical University, Taichung, Taiwan (CRREC-108-30).

The questionnaire was conducted through face-to-face interviews with each participant. The questionnaire included demographic information, socio-economic status, self-reported disease diagnoses (including SCZ and BPD), and Mini-Mental State Examination (MMSE). Birth cohort (decade of birth year) was stratified into five groups: <1950, 1950–1959, 1960–1969, 1970–1979, and ≥1980. Educational attainment was classified as illiteracy, self-study, elementary school, junior high school, senior high/vocational school, university/college, and master and above. The MMSE, the most commonly used tool for testing cognitive aging, was measured in subjects aged >60 years. Cognitive aging was defined as an MMSE score of <24. A total of 132,720 participants have joined the Taiwan Biobank, among which 33,741 have completed the first follow-up. Hence, the MMSE change during follow-up could be calculated.

### Genetic analysis and quality control

This study included 131,048 samples for whom genome-wide genotyping was performed. Genome-wide genotyping was performed using the custom Taiwan Biobank chips and run on the Axiom Genome-Wide Array Plate System (Affymetrix, Santa Clara, CA, USA); 27,716 participants were genotyped on the TWBv1 chip and 103,332 participants were genotyped on the TWBv2 chip. Quality control of the two batches was conducted separately before imputation, including the excluding variants with a call rate < 5%, minor allele frequency <0.001, and deviation from Hardy–Weinberg equilibrium with *P* < 1 × 10^–5^; 621,588 variants for TWBv1 and 504,241 variants for TWBv2 were kept. We used the 504 EAS panel from 1000 Genomes Project [36] and the 973 TWB panel from whole-genome sequencing in TWB participants as the reference panel to impute genotype with IMPUTE2 for the two chips separately (16,537,409 variants for TWBv1 and 16,211,759 variants for TWBv2), and then retained variants with imputation info score >0.7 (13,803,412 variants for TWBv1 and 13,572,189 variants for TWBv2). Most analyses excluded low quality imputated variants with imputation info lower than 0.4 or a more stringent threshold, and we excluded variants with imputation info lower than 0.7 to reach a balance between imputation accuracy and number of retained variants [37]. A total of 12,601,684 variants were available in both chips and kept for subsequent polygenic risk score (PRS) calculations. The chip version was adjusted for PRS-association analyses.

We excluded samples with a missing rate of more than 2% (*n* = 1), heterozygosity outliers exceeding 5 standard deviations (*n* = 700), duplicated samples (*n* = 1539), and non-EAS samples (*n* = 33), and 128,775 samples remained. To exclude cryptic relatedness, we estimated identity by descent (IBD) sharing coefficients, PI-HAT = probability (IBD = 2) + 0.5 × probability (IBD = 1), between any two participants and excluded one individual from a pair with PI-HAT > 0.1875 (*n* = 21969). A total of 106,806 unrelated individuals remained in the study.

### Polygenic risk score calculation

**Fig. 1:**
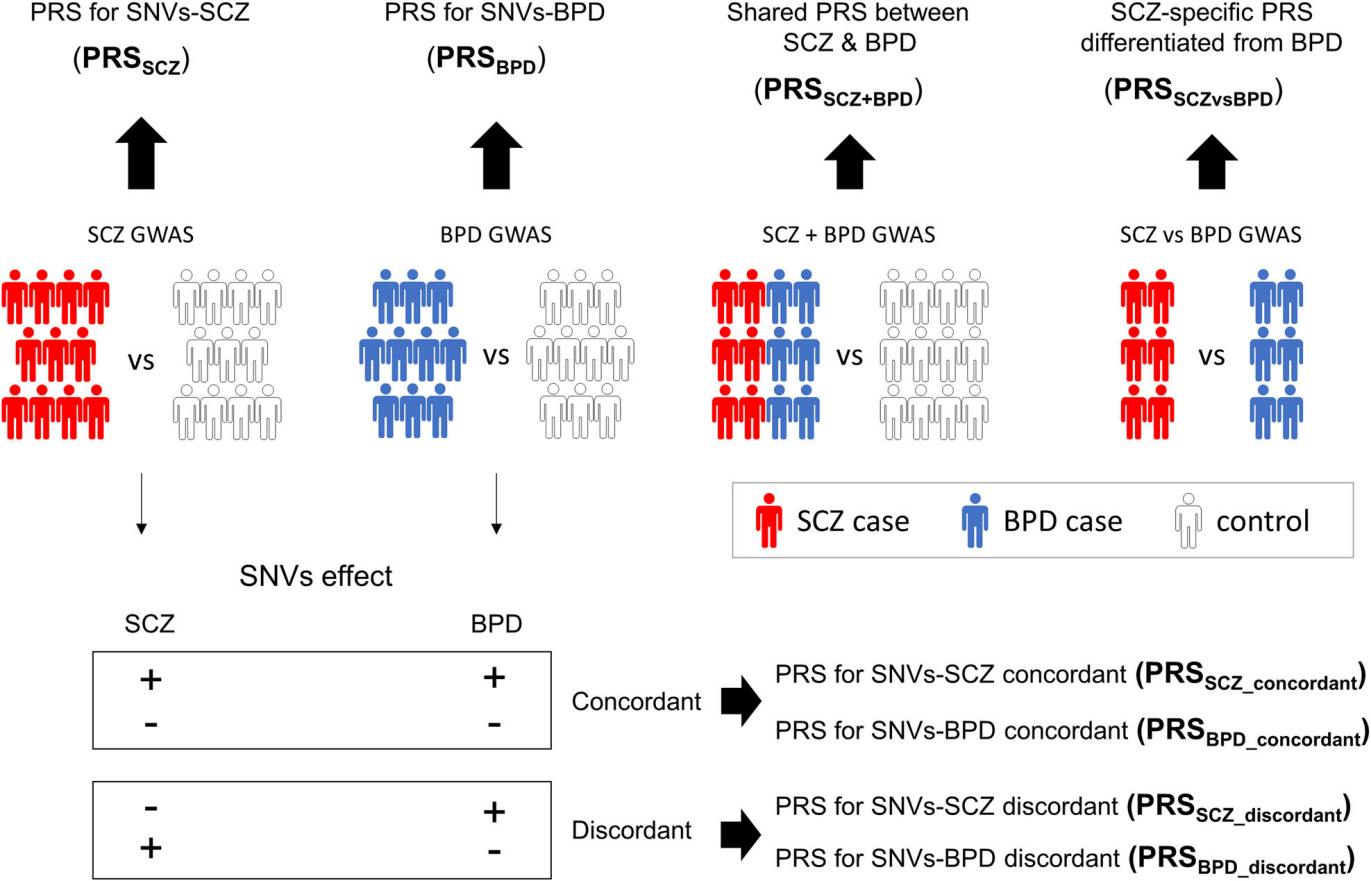
Overview of the eight polygenic risk scores (PRSs). SNV single nucleotide variant, GWAS genome-wide association study, SCZ schizophrenia, BPD bipolar disorder.

The PRS quantifies the cumulative additive effect of disease-associated single nucleotide variants (SNVs) across the genome. Using data from the latest Psychiatric Genomics Consortium (PGC) meta-analysis as discovery samples to identify susceptibility SNVs, we calculated eight PRSs for SNVs-SCZ (abbreviated as PRS_SCZ_), SNVs-BPD (PRS_BPD_), shared effect between SCZ and BPD (PRS_SCZ+BPD_), SCZ-specific effect differentiated from BPD (PRS_SCZvsBPD_), SNVs-SCZ concordant (PRS_SCZ_concordant_), SNVs-SCZ discordant (PRS_SCZ_discordant_), SNVs-BPD concordant (PRS_BPD_concordant_), and SNVs-BPD discordant (PRS_BPD_discordant_). Figure 1 presents an overview of the eight PRSs.

PRS_SCZ_ was derived from a core PGC GWAS for SCZ of 90 cohorts of European (EUR) and East Asian (EAS) ancestry, totaling 67,390 cases and 94,015 controls [38]. For comparison, we also calculated PRS for SNVs-SCZ-EAS (PRS_SCZ-EAS_) using the GWAS of EAS ancestry only, including 22,778 cases and 35,362 controls [39]. PRS_BPD_ was derived from GWAS for BPD with 41,917 cases and 371,549 controls [40]. To study shared genetic contribution to SCZ and BPD, PRS_SCZ+BPD_ was derived based on a GWAS of SCZ and BPD combined into a single phenotype, comparing 53,555 cases (33,426 SCZ patients + 20,129 BPD patients) with 54,065 controls [3]. To study divergent effects on SCZ and BPD, PRS_SCZvsBPD_ was derived based on a case-only GWAS comparing 23,585 patients with SCZ to 15,270 patients with BPD [3]. To capture the genetic heterogeneity of SCZ, we split PRS_SCZ_ into two scores based on sign-concordant [33] of the variants with SCZ [38] and BPD [40]; these variants that had concordant signs for SCZ and BPD (risk and risk or protective and protective on both traits) were combined into PRS_SCZ_concordant_, and the remaining variants that had discordant signs (risk and protective or protective and risk on both traits) were combined into PRS_SCZ_discordant_. In addition, we split PRS_BPD_ into PRS_BPD_concordant_ and PRS_BPD_discordant_.

All variants of the intersection of retained post-imputation variants and summary from a discovery sample were kept for subsequent PRS analyses. We derived PRS using Polygenic Risk Scores Continuous Shrinkage (PRS-CS), a polygenic prediction method inferring posterior effect sizes of susceptibility variants by utilizing a high-dimensional Bayesian regression framework and continuous shrinkage priors on susceptibility variant effect sizes, which have been shown robust to diverse underlying genetic architectures [41]; we used the 1000 Genomes Project phase 3 EUR samples as LD reference panel (–ref_dir = /ldblk_1kg_eur) and fixed global shrinkage parameter 0.01 (–phi=1e-2). Included variants for PRS calculation were detailed in supplementary file. For each subject in the Taiwan Biobank, the PRS was normalized to a Z-score. The variance explained by the association of the PRSs with SCZ and BPD was examined by logistic regression and the Nagelkerke’s pseudo-R^2^. To separate the genetic components of SCZ and BPD, participants with BPD were excluded when predicting SCZ, and vice versa. The correlations between the PRSs among participants with neither SCZ nor BPD were examined.

### Statistical analysis

The distribution of demographic factors, educational attainment, and MMSE was described by number and percentage, or mean and standard deviation (SD), according to the characteristics of the data. To explore the association with educational attainment, including five categories (from elementary school to master and above), an ordinal logistic regression model with adjustment for gender, age, birth cohort, batch version, and the top 20 population stratification dimensions was performed. To explore the association between PRS and MMSE scores and MMSE changes during follow-up, a linear regression model with adjustment for sex, age, birth cohort, educational attainment, batch version, and top 20 population stratification dimensions was performed. To test the association with cognitive deficits (MMSE score < 24), a logistic regression model with the same adjustments was performed. The disease course of SCZ/BPD and psychiatric treatments may result in lower educational attainment and worsened cognition, hence participants with SCZ or BPD were excluded from the PRS association test for educational attainment and MMSE.

Several model settings were tested to explore the complexity of polygenic liabilities for SCZ and BPD. Model 1 was used to test the independent effects of PRS_SCZ_ and PRS_BPD_. Model 2 tested the shared liabilities between SCZ and BPD (PRS_SCZ+BPD_). Model 3 tested the SCZ-specific risk contrast to BPD (PRS_SCZvsBPD_). Model 4 tested the split effect, including PRS_SCZ_concordant_, PRS_SCZ_discordant_, PRS_BPD_concordant_, and PRS_BPD_discordant_.

The overall significance level was set at 0.05. Four models were tested, hence the Bonferroni-corrected significance level was set at 0.05/4 = 0.0125. Nominal significance was defined if 0.0125 < *p* < 0.05 for an association test. All statistical analyses were performed using SAS 9.4.

## Results

Among 106,806 unrelated individuals, 192 reported having SCZ and 711 reported having BPD; 16 reported having both SCZ and BPD. The variance explained and the *p*-value of the association of the PRSs with SCZ or BPD are shown in Table 1. The PRS derived from SNVs-SCZ (PRS_SCZ_, EUR + EAS) explained more variance for SCZ in the Taiwan Biobank participants than that from SNVs-SCZ-EAS (PRS_SCZ-EAS_), thus it was used in the subsequent PRS association analyses. For predicting SCZ, PRS_SCZ_ and PRS_SCZ_concordant_ explained the most variance (4.32% and 4.10%, respectively), PRS_SCZ_discordant_ explained only 1.32%. PRS_BPD_concordant_ explained more variance in SCZ than PRS_BPD_ (1.86% vs. 1.14%), and PRS_BPD_discordant_ was not associated with SCZ. For predicting BPD, PRS_BPD_concordant_ led to a larger explained variance than PRS_BPD_ (0.48% vs. 0.39%), and PRS_SCZ_concordant_ led to a larger explained variance than PRS_SCZ_ (0.33% vs. 0.23%); PRS_SCZ_discordant_ was not associated with BPD. PRS_SCZ+BPD_ explained 1.64% and 0.38% in SCZ and BPD, respectively, while PRS_SCZvsBPD_ explained little variance in these two diseases.

**Table 1:** The association of polygenic risk score (PRS) for schizophrenia and bipolar disorder with the corresponding diseases in 106806 unrelated participants form the Taiwan Biobank data. Participants with BPD were excluded when predicting SCZ, and vice versa.

		Schizophrenia 176 cases & 105919 controls			Bipolar disorder 695 cases & 105919 controls		
PRS	# variants	OR	*p*-value	R^2a^ (%)	OR	*p*-value	R^2a^ (%)
SCZ	872,662	2.22	<0.001	4.32%	1.18	<0.001	0.23%
SCZ (EAS)	865,712	1.81	<0.001	2.39%	1.18	<0.001	0.23%
BPD	871,342	1.51	<0.001	1.14%	1.24	<0.001	0.39%
SCZBPD vs. CONT	867,670	1.63	<0.001	1.64%	1.24	<0.001	0.38%
SCZ vs.BPD	863,006	1.15	0.074	0.12%	0.92	0.024	0.06%
SCZ concordant	531,978	2.18	<0.001	4.10%	1.22	<0.001	0.33%
SCZ discordant	334,201	1.55	<0.001	1.32%	0.99	0.742	0.00%
BPD concordant	531,983	1.69	<0.001	1.86%	1.27	<0.001	0.48%
BPD discordant	334,204	0.94	0.369	0.03%	1.08	0.041	0.05%

^a^Increase in Nagelkerke pseudo R^2^ when adding the PRS into the model including gender, age, batch version, and 20 population stratification dimensions.

**Fig. 2:**
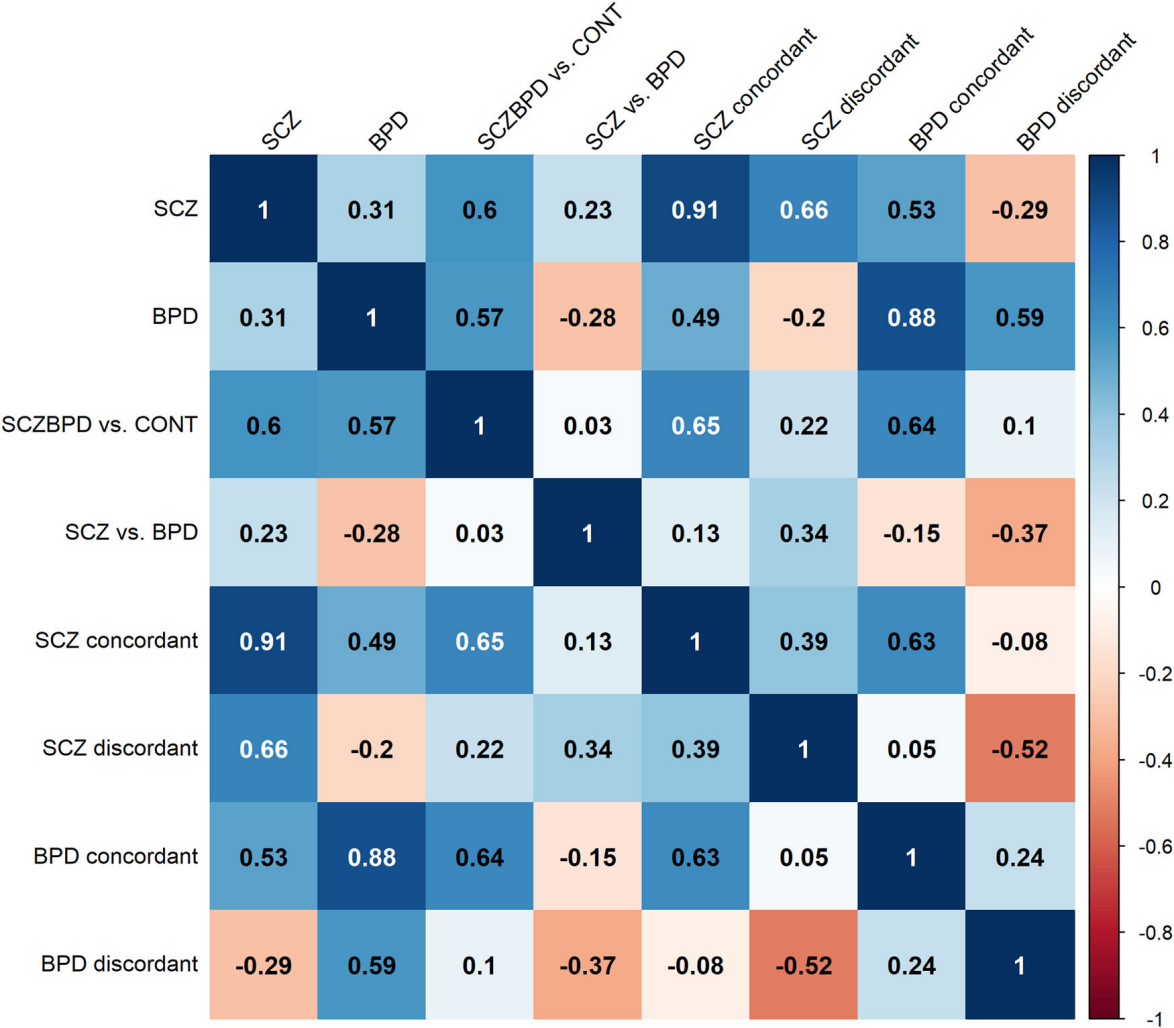
The correlations between the polygenic risk score (PRS) for schizophrenia (SCZ), bipolar disorder (BPD), SCZ and BPD shared risk, SCZ specific risk, SCZ concordant, SCZ discordant, BPD concordant, and BPD discordant among 105919 individuals without SCZ/BPD.

After excluding individuals with self-reported SCZ or BPD (*n* = 887), the correlations between the PRSs among 105,919 individuals without SCZ/BPD are shown in Fig. 2. PRS_SCZ_ had a moderate positive correlation with PRS_BPD_ (r = 0.31), a higher correlation with PRS_SCZ_concordant_ (r = 0.91) than with PRS_SCZ_discordant_ (0.66), a moderate positive correlation with PRS_BPD_concordant_ (r = 0.53), and a weak negative correlation with PRS_BPD_discordant_ (r = −0.29). PRS_BPD_ had a higher correlation with PRS_BPD_concordant_ (r = 0.88) than with PRS_BPD_discordant_ (r = 0.59), a moderate positive correlation with PRS_SCZ_concordant_ (r = 0.49), and a weak negative correlation with PRS_SCZ_discordant_ (r = −0.20). PRS_SCZ+BPD_ had a moderate positive correlation with PRS_SCZ_ (r = 0.60) and with PRS_BPD_ (r = 0.57). PRS_SCZvsBPD_ had a weak positive correlation with PRS_SCZ_ (r = 0.23) and a weak negative correlation with PRS_BPD_ (r = −0.28). There was a moderate positive correlation between PRS_SCZ_concordant_ and PRS_BPD_concordant_ (r = 0.63), and a moderate negative correlation between PRS_SCZ_discordant_ and PRS_BPD_discordant_ (r = −052.)

In the PRS association analyses for educational attainment and MMSE, individuals with self-reported SCZ or BPD (*n* = 887) and those with educational attainment being missing (*n* = 29), illiterate (*n* = 124), and self-study (*n* = 56) were excluded. A total of 105,710 samples remained, and the distribution of demographics is shown in Table 2. There were 67,120 females (63.5%), with a mean age of 49.9. The most common educational attainment was university (48.1%). The MMSE was available for 27,005 individuals aged ≥ 60 years. The mean MMSE score was 27.6, and 1663 participants (6.2%) were categorized as having cognitive deficits. Follow-up data for MMSE were available for 6194 individuals with a mean follow-up duration of 3.9 years (SD = 1.1 years), and the change in MMSE score was not statistically significant (mean = 0.2; SD = 3.4).

**Table 2:** Demographic characteristics among 105,710 unrelated individuals without schizophrenia and bipolar disorder from the Taiwan Biobank data.

Variable	*n* (%)
Gender	
Female	67,120 (63.5)
Male	38,590 (36.5)
Age at baseline	
30–39	23,239 (22.0)
40–49	26,873 (25.4)
50–59	28,579 (27.0)
60–70	27,019 (25.6)
Mean ± SD	49.9 ± 10.9
Birth cohort	
<1950	5911 (5.6)
1950–1959	28,722 (27.2)
1960–1969	29,990 (28.4)
1970–1979	25,677 (24.3)
≥1980	15,410 (14.6)
Educational attainment	
Elementary school	4673 (4.4)
Junior high school	7539 (7.1)
Senior high/Vocational school	30,754 (29.1)
University/College	50,794 (48.1)
Master and above	11,950 (11.3)
MMSE at baseline (*n* = 27,005)	
<24	1663 (6.2)
≥24	25,342 (93.8)
Mean ± SD	27.6 ± 3.8
MMSE Change (*n* = 6194)	
Mean ± SD	0.2 ± 3.4

*SD* standard deviation.

The results of the association of polygenic liabilities for SCZ and BPD with educational attainment and MMSE under different models are shown in Table 3. For ordinal education, both PRS_SCZ_ (OR = 1.044, *p* < 0.001) and PRS_BPD_ (OR = 1.021, *p* = 0.001) were associated with higher educational attainment. In split model, the two concordant PRSs were, but the two discordant PRSs were not, positively with educational attainment. Shared risk between SCZ and BPD (PRS_SCZ+BPD_) was positively associated with educational attainment (OR = 1.053, *p* < 0.001), whereas SCZ-specific risk (PRS_SCZvsBPD_) was negatively associated with educational attainment, although it only reached nominal significance (OR = 0.986, *p* = 0.017).

**Table 3:** Association of polygenic risk score (PRS) for schizophrenia and bipolar disorder with MMSE and educational attainment.

	Ordinal education (*n* = 105,710)		MMSE (*n* = 27,005)		MMSE < 24 (*n* = 27,005)		MMSE change (*n* = 6194)	
PRS	aOR (95% CI)^c^	*P*	Beta (95% CI)^a^	*P*	aOR (95% CI)^a^	*P*	Beta (95% CI)^b^	*P*
Model 1: independent effect								
SCZ	1.044 (1.031–1.057)	<0.001	−0.069 (−0.115, −0.023)	0.003	1.066 (1.008–1.127)	0.025	−0.045 (−0.135, 0.046)	0.334
BPD	1.021 (1.009–1.033)	0.001	0.054 (0.009, 0.010)	0.020	0.917 (0.867–0.970)	0.003	0.067 (−0.023, 0.158)	0.146
Model 2: SCZ & BPD shared								
SCZBPD vs. CONT	1.053 (1.041–1.065)	<0.001	−0.023 (−0.067, 0.020)	0.294	1.019 (0.967–1.075)	0.477	0.038 (−0.049, 0.124)	0.395
Model 3: SCZ-specific								
SCZ vs.BPD	0.986 (0.975–0.998)	0.017	−0.037 (−0.080, 0.006)	0.092	1.015 (0.963–1.069)	0.589	−0.125 (−0.210, −0.040)	0.004
Model 4: split effect								
SCZ concordant	1.030 (1.013–1.047)	0.001	−0.101 (−0.164, −0.038)	0.002	1.088 (1.007–1.175)	0.033	−0.062 (−0.189, 0.064)	0.335
SCZ discordant	1.011 (0.996–1.026)	0.142	0.035 (−0.021, 0.090)	0.221	0.987 (0.922–1.056)	0.707	−0.019 (−0.129, 0.090)	0.730
BPD concordant	1.022 (1.006–1.038)	0.007	0.073 (0.013, 0.133)	0.017	0.918 (0.853–0.989)	0.024	0.082 (−0.038, 0.202)	0.180
BPD discordant	1.001 (0.987–1.015)	0.894	0.049 (−0.005, 0.102)	0.073	0.950 (0.890–1.014)	0.122	−0.004 (−0.108, 0.101)	0.947

^a^Adjustment for gender, age, birth cohort, education level, TWB (chip version) and first 20 population stratification dimensions.

^b^Adjustment for gender, age, birth cohort, education level, duration of follow-up, TWB (chip version) and first 20 population stratification dimensions.

^c^Adjustment for gender, age, birth cohort, TWB (chip version), and first 20 population stratification dimensions.

For cognitive aging, PRS_SCZ_ was associated with lower MMSE (beta in per SD increase in PRS = -0.069, *p* = 0.003), while PRS_BPD_ was nominally significant associated with higher MMSE (beta = 0.054, *p* = 0.020). The split model suggested that the concordant PRSs, but not the discordat PRSs, were associated with MMSE. Both shared risks between SCZ and BPD, PRS_SCZ+BPD_, and SCZ-specific PRS, PRS_SCZvsBPD_, were not associated with baseline MMSE.

When analyzing the MMSE as a binary outcome, the presence or absence of cognitive deficits, the results remained similar. PRS_SCZ_ was nominally significant associated with a higher risk of cognitive deficits (odds ratio [OR] per SD increase in PRS = 1.066, *p* = 0.025), while PRS_BPD_ was associated with a lower risk of cognitive deficits (OR = 0.917, *p* = 0.003).

For MMSE changes during follow-up, PRS_SCZ_ and PRS_BPD_ were not associated with MMSE change. PRS_SCZvsBPD_ (beta = -0.125, *p* = 0.004) predicted decreases in the MMSE scores.

## Discussion

Using a large collection of community samples from the Taiwan Biobank, this study explored the polygenic risks of SCZ and BPD on educational attainment and cognitive aging using individual genotype and phenotype data. We found that both polygenic liabilities for SCZ and BPD were positively associated with high educational attainment, which was mainly determined by the shared or concordant polygenic liabilities for SCZ and BPD. The SCZ-specific risk was inversely associated with educational attainment. In cognitive aging, polygenic liability for SCZ was associated with an increased risk of cognitive aging; however, polygenic liability for BPD had a reduced risk. The SCZ-specific polygenic risk was associated MMSE decline during follow-up period. The concordant and discordant parts of SCZ and BPD polygenic liabilities have contrasting association with educational attainment and cognitive aging.

The findings that PRS_SCZ_ was associated with BPD, and PRS_BPD_ was associated with SCZ, suggested that SCZ and BPD share a genetic architecture. Compared to PRS_BPD_, PRS_BPD_concordant_ explained a higher variance in SCZ. For predicting BPD, PRS_BPD_concordant_ explained more variance than PRS_BPD_ and PRS_SCZ_concordant_ explained more variance than PRS_SCZ_. Our results showed that splitting the PRS improved the prediction and provided genetic evidence that SCZ and BPD were not genetically homogeneous, which was further supported by a moderate negative correlation between PRS_SCZ_discordant_ and PRS_BPD_discordant_.

The finding that PRS_SCZ_ and PRS_BPD_ were positively associated with high educational attainment is consistent with previous studies demonstrating that there are overlapping genes between education and SCZ [42] or BPD [21]. However, the positive association of polygenic liability for SCZ with education was mainly attributed to the shared part with BPD as well as the observed positive genetic correlation between SCZ and BPD. We found that the SCZ-specific risk was negatively associated with education. Our findings are consistent with a recent study using linkage disequilibrium score regression, showing that both SCZ and BPD genetic backgrounds have a positive but SCZ-specific genetic background that has a negative genetic correlation with educational attainment [23].

Education and cognitive performance have a shared genetic basis [43]. Higher educational attainment could allow individuals to preserve better cognitive performance in late life [44]. However, cognitive aging was different from educational attainment in the associations with the genetic liability of SCZ and BPD. We found that the polygenic liability of SCZ was associated with an increased risk of cognitive deficits. Our finding was consistent with previous epidemiological studies, which showed that patients with SCZ had a higher risk for dementia [20]. Furthermore, one study found that first-degree relatives of probands with frontotemporal dementia have an increased risk of developing SCZ [45], indicating a shared genetic risk between SCZ and cognitive decline. In addition, several studies have demonstrated that the polygenic risk of SCZ is associated with poor cognitive performance, especially among older adults [46–48]. We expected the inconsistency between educational attainment and cognitive aging could be explained by the SCZ-specific risk; however, the results were against our expectations. No association between cognitive aging and SCZ-specific risk was noted. However, among 6,194 patients who had repeated MMSE measurements, we found that SCZ-specific liability was associated with MMSE decline. Cognitive aging might be determined by non-cognition factors, such as underlying cardiovascular diseases [49], which are commonly noted among individuals with SCZ [50].

Although previous epidemiological studies have also demonstrated that patients with BPD have a higher risk of developing dementia [51] and some studies demonstrated polygenic liability for BPD might be associated with poor cognitive performance [52], we found that the polygenic liability of BPD was related to a high MMSE score. Our finding was in line with a previous study that showed that patients with BPD have higher cognitive abilities compared to controls [53].

The PRS only considers additive effect of common variants and does not consider their interaction effect and the effect of rare variants, hence it does not fully capture the genetic vulnerability. In addition, for genetic architecture may differ across populations, using cross-ancestry GWAS results to calculate PRS may lead to a low prediction [54]. When using European ancestry as discovery samples, the prediction performance in target samples of Asian or African ancestry was 37–78% lower compared with that in target samples of European ancestry [55]. The discovery sample used in this study were mainly from the European ancestry, and we applied the PRS-CS for improving cross-population polygenic prediction in the Asian samples. We noted that the PRSscz and PRS_BPD_ only explained 4.3% and 0.4%, respectively, of the corresponding disease status in the Taiwan Biobank samples, in which the prediction performance of the PRS was much smaller than in PGC discovery samples [38, 40]. Our study is advantageous in that it conducted a large-scale population-based genetic investigation utilizing a large collection of non-European samples. We used the most well-powered GWAS from PGC as discovery samples to identify susceptibility variants. However, the discovery sample for shared PRS between SCZ and BPD and SCZ-specific PRS [3] is relatively old and its sample is about half of the combined SCZ and BPD latest GWAS [38, 40]. Further SCZ + BPD GWAS with enlarged sample will improve PRS prediction power. We applied the split PRS method based on sign-concordant [33] of the variants of SCZ and BPD to explore the two disorders’ genetic homogeneity and heterogeneity, further investigation could consider other methods, e.g., genomic structural equation modelling [56–58], to examine the joint genetic architecture.

This study has several limitations. First, the disease status of patients with SCZ and BPD was obtained by retrospective self-reporting, which may have led to recall bias and resulted in misclassification and underestimation of the prevalence of diseases. A recent study [59] has evaluated the accuracy of the self-reported disease status with the ICD diagnosis in the Taiwan National Health Insurance Research Database. The data show that the tetrachoric correlations for SCZ and BPD were 0.95 and 0.72, respectively. Second, we used the MMSE to detect cognitive aging; however, the MMSE is affected by education level. Patients who are well-educated perform better in the MMSE. In our study, we found that PRSscz and PRS_BPD_ were positively associated with educational attainment. Thus, the adverse effects of PRSscz on cognitive aging might be underestimated. Third, the Taiwanese Biobank participants may have been biased towards being healthy. Individuals with high genetic liabilities for psychiatric disorders and poor mental health had a low chance of being included in our analyses. Fourth, we only used individuals of Asian ancestry. Further investigation is warranted to determine whether our findings can be generalized to other populations. Most large-scale GWAS have been performed in individuals of EUR populations, with only a few in individuals of EAS populations, e.g., SCZ. However, the PRS derived from matched ancestry (PRS_SCZ_EAS_) did not lead to a better prediction of SCZ in the Taiwan Biobank samples than the PRS derived from EUR + EAS ancestries (PRS_SCZ_) with a much larger sample size. In addition to the issue of cross-ancestry PRS prediction, the sample size for the discovery sample is also crucial. Further large-scale genetic research in individuals of diverse ancestries is needed.

This study provides evidence for the contrasting effects of polygenic liabilities in SCZ and BPD on cognitive aging. Although polygenic liabilities for both SCZ and BPD were independently associated with higher educational attainment, SCZ-specific polygenic liability was associated with lower educational attainment. The concordant and discordant parts of polygenic liabilities have contrasting association with educational attainment and cognitive aging. Our findings partially support the hypothesis that the heterogeneity of SCZ and the positive association of polygenic liability for SCZ with education might be attributed to the shared part with BPD.

## Supplementary information

Variants selected for each PRS
